# Exploring the Relationship Between Behavioral Inhibition and Approach Systems and Alcohol‐Related Outcomes in People With Alcohol Use Disorder

**DOI:** 10.1002/brb3.70944

**Published:** 2025-10-20

**Authors:** Madeline E. Crozier, Lorenzo Leggio, Mehdi Farokhnia

**Affiliations:** ^1^ Clinical Psychoneuroendocrinology and Neuropsychopharmacology Section, Translational Addiction Medicine Branch, National Institute On Drug Abuse Intramural Research Program and National Institute On Alcohol Abuse and Alcoholism Division of Intramural Clinical and Biological Research National Institutes of Health Baltimore and Bethesda Maryland USA; ^2^ Center For Alcohol and Addiction Studies, Department of Behavioral and Social Sciences, School of Public Health Brown University Providence Rhode Island USA; ^3^ Division of Addiction Medicine, Department of Medicine, School of Medicine Johns Hopkins University Baltimore Maryland USA; ^4^ Department of Neuroscience Georgetown University Medical Center Washington DC USA; ^5^ Department of Mental Health, Johns Hopkins Bloomberg School of Public Health Johns Hopkins University Baltimore Maryland USA

**Keywords:** addiction, alcohol, behavioral approach, behavioral inhibition, BIS/BAS

## Abstract

**Background:**

The Behavioral Inhibition System (BIS) and the Behavioral Approach System (BAS) are two core motivational systems linked to addictive behaviors. Understanding the biobehavioral mechanisms and correlates of Alcohol Use Disorder (AUD), including BIS/BAS, could lead to improved strategies for prevention, diagnosis, and treatment.

**Methods:**

Using baseline data from five clinical studies, we conducted secondary analyses to explore the link between BIS/BAS and alcohol‐related outcomes in people with AUD (*N* = 94). We hypothesized that lower BIS and higher BAS scores would be associated with more severe alcohol use, obsessive thoughts, and compulsive behaviors toward alcohol. In additional post‐hoc analyses, we also explored the mediating effects of anxiety and depression in this regard.

**Results:**

Higher BIS scores were associated with higher severity of alcohol use and more obsessive‐compulsive drinking behaviors, as respectively measured by the Alcohol Use Disorder Identification Test (AUDIT) and the Obsessive‐Compulsive Drinking Scale (OCDS). Anxiety (Spielberger State‐Trait Anxiety Inventory) and depression (Montgomery–Asberg Depression Rating Scale) significantly mediated the positive associations between BIS scores and AUDIT/OCDS. No significant associations were found between BAS scores and alcohol‐related measures.

**Conclusions:**

These findings suggest that, in this sample of middle‐aged people with AUD, a heightened BIS leads to more severe alcohol use, and this relationship is mediated by anxiety and depressive symptoms. Further prospective research in adults with AUD and varying levels of alcohol use is necessary to better understand the relationship between BIS/BAS and alcohol‐related outcomes.

## Graphical abstract

This study examined behavioral inhibition (BIS) and approach (BAS) systems in middle‐aged individuals with alcohol use disorder. Higher BIS scores were associated with more severe alcohol use and greater obsessive‐compulsive drinking behaviors, while BAS scores showed no significant associations. Post‐hoc mediation analyses showed that anxiety and depressive symptoms explained much of the relationship between BIS and alcohol‐related outcomes.

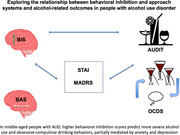

## 1 Introduction

Alcohol use disorder (AUD) is a leading cause of morbidity and mortality worldwide (GBD 2016 Alcohol Collaborators 2018). Effective prevention, diagnosis, and treatment of AUD require a better understanding of the pathophysiology of this disorder (Witkiewitz et al. 2019). Several biobehavioral and psychosocial factors have been implicated in the development and progression of AUD, including those related to motivations to seek and consume alcohol. The behavioral inhibition system (BIS) and the behavioral approach system (BAS) are two core motivational systems linked to alcohol use and addictive behaviors (Studer et al. 2016; Franken et al. 2006; Hamilton et al. 2012).

The BIS is thought to be activated by conflicting stimuli and is responsible for the defensive approach to dangerous or unpleasant situations via, e.g., noradrenergic and serotonergic activity in the septo‐hippocampal system (Meyer et al. 1999). Activation of this system tends to elicit fear and anxiety (Papachristou et al. 2018). The BAS is thought to be activated by reward‐related cues or escape from punishment, mainly via mesolimbic dopamine pathways (Del Giudice 2023). As described by Carver and White (1994), the BAS can be characterized by three components: drive, fun‐seeking, and reward responsiveness (Carver and White 1994). Drive refers to the persistence in achieving goals; fun‐seeking refers to the desire for new rewards and taking on spur‐of‐the‐moment opportunities; and reward responsiveness refers to the positive response to a reward (Vecchione et al. 2021). It is generally thought that a heightened BAS is associated with high‐risk behaviors/outcomes, including AUD and other substance and non‐substance addictive disorders, whereas a heightened BIS is thought to play a protective role (Black et al. 2014). The BIS/ BAS Scale was developed to measure individual differences in how strongly each system is activated in response to different situations. The BIS measurement characterizes a person's level of caution and desire to avoid adverse outcomes, while the BAS measurements characterize a person's impulsivity and desire to elicit a reward response (Carver and White 1994).

Previous studies have investigated the relationship of BIS/BAS and addictive behaviors, including alcohol use, and suggest that a stronger BAS, as indicated by higher BAS scores, is associated with higher rates of problematic drinking (Franken et al. 2006; Hamilton et al. 2012; Rieser et al. 2019; Sprah and Novak 2008). The BIS, on the other hand, has been less studied, and findings have been mixed. In three different studies with college students, lower BIS scores predicted increased alcohol use in terms of quantity and frequency, resulting in heavy drinking (Kimbrel et al. 2007; O'Connor et al. 2009; Pardo et al. 2007). However, another study among college students found the opposite, where higher BIS scores were associated with increased alcohol consumption (Wardell et al. 2011). Two other studies in young adults found no significant associations between BIS scores and alcohol use (Hundt et al. 2008; Knyazev 2004). A potential factor contributing to these heterogeneous results could be the role of other psychosocial variables, such as mood and anxiety. A more active BIS is typically associated with higher anxiety (Levita et al. 2014; Oniszczenko 2025). Anxiety is often a trigger that leads to more alcohol drinking and there is high comorbidity between anxiety disorders and AUD (Kushner et al. 2000). On the other hand, a more active BIS may protect against problematic drinking because this system can influence people to be more cautious, and alcohol can have undesirable outcomes, such as a lost sense of control or negative health problems (Wardell et al. 2011; Knyazev 2004). Therefore, it is plausible to hypothesize that anxiety may mediate the relationship between the BIS and alcohol‐related outcomes, which may explain, at least in part, the mixed findings reported in the previous studies. In addition, the link between AUD and depressive disorders is well established (McHugh and Weiss 2019; Stephen Rich and Martin 2014), and previous research also suggests that higher BIS scores are associated with depression (Kasch et al. 2002; Li et al. 2015). Accordingly, depression may also mediate the relationship between BIS and alcohol‐related outcomes.

To expand the existing literature, the aim of this study was to examine the relationship between the behavioral inhibition/approach systems and alcohol‐related outcomes in adult individuals diagnosed with AUD—a novel and clinically relevant aspect, especially given that most prior studies focused on younger individuals and college students who typically have a shorter lifetime drinking history, lower prevalence and severity of AUD, and a different drinking pattern (e.g., binge drinking). We hypothesized that a less active BIS and a more active BAS would be associated with more severe alcohol use, more obsessive and compulsive thoughts and actions towards alcohol, and higher alcohol consumption. Following our primary results, we further hypothesized that anxiety and/or depression would indirectly mediate the relationship between the BIS and alcohol‐related outcomes.

## 2 Methods

### 2.1 Setting and Participants

This secondary study was pre‐registered at https://osf.io/2arnd, and data were derived from all alcohol‐related human laboratory studies conducted and completed at the National Institutes of Health Intramural Research Program (NIH IRP) Clinical Psychoneuroendocrinology and Neuropsychopharmacology Section to date, with no exception (NCT01751386; NCT01779024; NCT02039349; NCT02707055; NCT03152760). All studies were approved by the appropriate Institutional Review Board and conducted at the NIH Clinical Center, Bethesda, MD, USA. Informed consent was obtained, and participants received compensation. The inclusion/exclusion criteria of the five parent studies are presented in the supplement (Appendices S1–S5). All participants were 21 years or older and had a current diagnosis of AUD according to the Diagnostic and Statistical Manual of Mental Disorders, Fourth (DSM‐IV) or Fifth (DSM‐5) Edition, a negative urine drug test, and no significant medical or psychiatric comorbidities. All assessments for the current study were conducted at baseline, prior to the initiation of any intervention, to minimize potential confounding by study participation, procedures, or interventions.

### 2.2 Assessments

*Behavioral Inhibition System/Behavioral Approach System (BIS/BAS) Scale*: The BIS/BAS scale was developed to measure individual differences in the sensitivity of behavioral inhibition and approach systems. The scale consists of 24 statements (20 main items plus 4 fillers) ranked on a scale from 1 (“very true for me”) to 4 (“very false for me”). The questionnaire includes statements such as “I feel pretty worried or upset when I think or know somebody is angry at me” to measure the BIS and statements such as “when I want something, I usually go all out to get it” to measure the BAS. The scale is categorized into four subscales: BIS, BAS Drive, BAS Fun Seeking, and BAS Reward Responsiveness; a single BAS score can also be calculated by averaging the three BAS subscale scores (Carver and White 1994). The scale demonstrated acceptable internal consistency in this sample, as indicated by Cronbach's *α*’s: BIS (7 items, *α* = 0.72), BAS Drive (4 items, *α* = 0.83), BAS Fun Seeking (4 items, *α* = 0.76), BAS Reward Responsiveness (5 items, *α* = 0.67), BAS all items (13 items, *α* = 0.86).

*Alcohol Use Disorders Identification Test (AUDIT)*: The AUDIT is a 10‐item multiple‐choice questionnaire designed to assess the severity of alcohol use over the past year. It evaluates alcohol consumption patterns, drinking behaviors, and problems associated with alcohol use. Total scores range from 1 to 40, with scores of 1–7 suggesting low‐risk drinking, 8–14 hazardous/harmful use, and 15–40 potential alcohol dependence (moderate to severe AUD). The AUDIT includes three subscales: Consumption (C), Dependence (D), and Hazardous (H) (Babor et al. 1992).

*Obsessive‐Compulsive Drinking Scale (OCDS)*: The OCDS measures obsession and compulsion related to alcohol craving and drinking over the course of the past week. The assessment consists of 14 questions scored on a scale of 0–4. Total scores range from 0–56, with higher scores indicating more thoughts and actions related to alcohol obsession and compulsion. The OCDS is divided into two subscales: The Obsessive Drinking Scale and Compulsive Drinking Scale (Anton 2000).

*Alcohol Timeline Follow‐Back (TLFB)*: The TLFB uses a calendar‐based approach that begins the day before the assessment and typically covers the previous 30 or 90 days. During an interview with a trained researcher, participants report the number of standard drinks they had each day, where one standard drink in the US equals 14 grams of alcohol—approximately 12 oz of beer, 5 oz of wine, or 1.5 oz of spirits. These data are then used to generate various summary measures. For the present analysis, two variables were examined: The average number of standard drinks consumed on drinking days and the number of heavy drinking days over the past 90 days. Heavy drinking days were defined as consuming more than 3 or 4 drinks in a day for females or males, respectively (Sobell and Sobell 1992; Sobell et al. 1979; National Institute of Alcohol Abuse and Alcoholism [NIAAA] 2023).

*Spielberger State‐Trait Anxiety Inventory (STAI)*: The STAI measures overall anxiety levels and can be collected as both a state and a trait assessment. The trait version of STAI was used for this study as a relatively stable characteristic. The inventory consists of 20 questions that are self‐reported on a 4‐point Likert scale. Total scores range from 20–80, with a score of 20–37 indicating no or low anxiety, 38–44 moderate anxiety, and 45–80 high anxiety (Spielberger et al. 1983).

*Montgomery‐Asberg Depression Rating Scale (MADRS)*: The MADRS is a 10‐item scale designed to measure the severity of depressive symptoms. Each item is ranked on a scale from 0 (no agreement with the statement) to 6 (strong agreement with the statement). Total scores range from 0–60, with a score of 0–6 indicating no depression, 7–19 mild depression, 20–34 moderate depression, and 35–60 severe depression (Montgomery and Asberg 1979).

### 2.3 Analyses

Study variables and demographic characteristics of the study sample were summarized using descriptive statistics. The Kolmongorov–Smirnov test was used to examine normal distribution. Most data were not normally distributed; therefore, we used nonparametric Spearman correlations to explore bivariate correlations between BIS/BAS scores and alcohol‐related measures, including AUDIT, OCDS, and TLFB. Based on the results, separate regression analyses were then performed with BIS scores as an independent variable and AUDIT and OCDS scores as the outcome, with and without controlling for demographic variables. To test the Joint Subsystems Hypothesis (Wardell et al. 2011; Corr 2002), we also ran regression models including the interaction between BIS and each BAS variable (the three subscales and average score). Considering reported differences between treatment‐seeking and non‐treatment‐seeking individuals with AUD (Rohn et al. 2017; Ray et al. 2017; Lee et al. 2019; Haass‐Koffler et al. 2020), we also ran regression models including the interaction between BIS and treatment‐seeking status. *R* ^2^ values were calculated to determine the proportion of variance in the outcomes predicted by the independent variables, with values ranging from 0 to 1. Variance inflation factor (VIF) was also calculated to test for multicollinearity in the regression models; VIF scores higher than 5 generally indicate problematic multicollinearity.

Post‐hoc mediation analyses were conducted using Model 4 with the PROCESS Macro (Version 4.2) in Statistical Package for the Social Sciences (SPSS). These analyses stemmed from our primary analyses and tested indirect effects of STAI and MADRS as potential mediators of the relationship between BIS scores and total AUDIT/OCDS, utilizing standardized path coefficients. Since age was a significant predictor of STAI (see Table S5), mediation analyses with STAI were controlled for age. Significance testing was achieved with 5000 bootstrap samples and the absence of 0 in the 95% confidence intervals (CIs).

Analyses were done with SPSS version 28 (IBM, Armonk, NY, USA), and a *p* < 0.05 (two‐tailed) was considered statistically significant.

## 3 Results

### 3.1 Demographic Characteristics and Summary Statistics

Table 1 presents the mean, standard deviation (SD), and range for continuous variables and the number and percent for categorical variables. Adult participants with a current diagnosis of AUD and complete BIS/BAS, AUDIT, OCDS, and TLFB data were included in the study. Of one hundred forty‐seven participants enrolled in the five parent studies, fourteen were excluded for no current AUD diagnosis, and twenty‐five were excluded for missing data on at least one of the scales. Twelve participants had participated in more than one parent study and, therefore, only their most recent data were used. Two participants were removed for having outlying TLFB data (more than 3500 drinks in 90 days). Therefore, a total of ninety‐four participants were included in the present analysis (Figure S1). Participants were predominantely male (78.7%) and, on average, 46.0 years old (SD = 10.8). Around 45% of the participants were seeking treatment for AUD.

**TABLE 1:** Demographic variables and summary statistics.

Variable		*n*	%	Mean	Standard deviation	Range
**Demographics**	**Age,** years			46.0	10.8	22–69
	**Sex**					
	‐ Male	74	78.7			
	‐ Female	20	21.3			
	**Race**					
	‐ White/European American	42	44.7			
	‐ Black/African American	47	50.0			
	‐ Asian	3	3.2			
	‐ Multiracial	1	1.1			
	‐ Unknown	1	1.1			
	**Ethnicity**					
	‐ Hispanic/Latino	0	0			
	‐ Not Hispanic/Latino	92	97.8			
	‐ Unknown	2	2.2			
	**Years of Education**			12.9	3.8	2–22
	**BMI,** kg/m^2^			27.0	4.4	17.5–39.0
	Current AUD Diagnosis*	94	100			
	**Seeking TAreatment for AUD**	43	45.7			
	**Current Cigarette Smoker**	57	60.6			
**Assessments**	**BIS Score**			18.9	3.5	7–27
	**BAS Drive Score**			11.5	2.4	6–16
	**BAS Fun‐Seeking Score**			11.9	2.2	6–16
	**BAS Reward Responsiveness Score**			16.8	1.9	11–20
	**TLFB Average Drinks Per Drinking Day**			12.3	8.5	1.9–40.0
	**TLFB Heavy Drinking Days**			61.3	27.5	1–90
	**AUDIT score**			24.0	7.6	1–36
	**OCDS score**			18.0	8.1	0–40
	**STAI score**			45.2	10.1	22–73
	**MADRS score**			9.5	8.5	0–35

Abbreviations: AUD, Alcohol Use Disorder, BIS, Behavioral Inhibition System; BAS, Behavioral Approach System; TLFB, Timeline Follow‐Back; AUDIT, Alcohol Use Disorders Identification Test; OCDS, Obsessive‐Compulsive Drinking Scale; STAI, Spielberger State‐Trait Anxiety Inventory; MADRS, Montgomery–Asberg Depression Rating Scale.

*AUD diagnosis was determined by the Structured Clinical Interview for DSM‐5 (SCID 5) or by alcohol dependence or alcohol abuse according to the Structured Clinical Interview for DSM‐4 (SCID IV).

### 3.2 Associations Between BIS/BAS Scores and Alcohol‐Related Measures

Table 2 presents the results of bivariate correlations between our variables of interest. BIS scores positively correlated with Total OCDS scores (ρ = 0.25, p = 0.01) and had a trend‐level positive association with total AUDIT scores (ρ = 0.179, p = 0.08). There were no significant correlations between the BAS variables and any of the alcohol‐related variables. A full table depicting correlations with subscales can be found in the supplement (Appendix S6), highlighting the Compulsive Drinking subscale of OCDS and AUDIT‐H as the main contributors of the abovementioned associations with BIS.

**TABLE 2:** Bivariate correlations of alcohol‐related variables with BIS/BAS scores.

	TLFB average drinks per drinking day	TLFB heavy drinking days	Total AUDIT score	Total OCDS score
**BIS score**				
Spearman correlation coefficient (*ρ*)	−0.127	−0.044	0.179	0.25
*p* value	0.222	0.676	0.085	0.015
**BAS drive score**				
Spearman correlation coefficient (*ρ*)	−0.072	−0.045	0.058	0.112
*p* value	0.493	0.667	0.577	0.282
**BAS fun‐seeking score**				
Spearman correlation coefficient (*ρ*)	−0.049	−0.049	0.012	0.071
*p* value	0.640	0.638	0.909	0.497
**BAS reward responsiveness score**				
Spearman correlation coefficient (*ρ*)	0.002	0.007	0.002	−0.124
*p* value	0.987	0.946	0.984	0.234
**BAS average score**				
Spearman correlation coefficient (*ρ*)	−0.033	0.000	0.068	0.064
*p* value	0.753	0.997	0.518	0.541

Abbreviations: AUDIT, Alcohol Use Disorders Identification Test; BAS, Behavioral Approach System Scale, BIS, Behavioral Inhibition System Scale; OCDS, Obsessive‐Compulsive Drinking Scale; TLFB, Timeline Follow‐Back.

Table 3 summarizes the results of the multiple regression analyses, with full results presented in Appendix S7. All VIFs were under 5, indicating no significant multicollinearity. BIS score was a significant predictor of Total AUDIT and Total OCDS scores, with and without controlling for demographic variables. When controlling for demographics, the regression models explained 11.7% of the variance in Total AUDIT scores and 12.5% of the variance in OCDS scores.

**TABLE 3:** Summary of multiple regression analyses with BIS score as an independent variable.

	Not controlling for demographics					Controlling for demographics^*^				
Outcome	*B*	Standard error	*β*	*t*	*p*	*B*	Standard error	*β*	*t*	*p*
**AUDIT score**	0.484	0.222	0.222	2.180	0.032	0.590	0.242	0.270	2.433	0.017
**OCDS score**	0.544	0.235	0.235	2.318	0.023	0.769	0.256	0.332	3.006	0.003

Abbreviations: AUDIT, Alcohol Use Disorders Identification Test; OCDS, Obsessive‐Compulsive Drinking Scale.

* Demographics that were controlled for include: age, sex, race, years of education, BMI, and smoking status.

Regression models testing BIS × BAS interactions on Total AUDIT or Total OCDS scores did not show any significant interactions between BIS and BAS scores (*data not shown*). Similarly, treatment‐seeking status did not moderate the association between BIS and Total AUDIT or Total OCDS scores as indicated by no significant interaction effects (*data not shown*).

### 3.3 Mediation Analyses With Anxiety and Depression Measures

**FIGURE 1:**
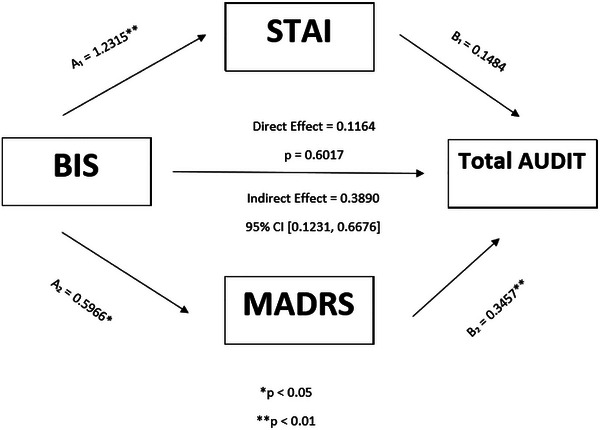
Parallel mediation with STAI and MADRS as mediators of the relationship between BIS and Total AUDIT (controlling for age).

Figure 1 depicts the parallel mediation effects of STAI and MADRS scores on the relationship between BIS and Total AUDIT scores. A significant indirect effect mediated by STAI and MADRS scores, while controlling for age, was found. Specifically, BIS scores were significantly associated with STAI (A_1_ = 1.23, 95% CI [0.67, 1.79], *p* < 0.01) and MADRS (A_2_ = 0.59, 95% CI [0.08, 1.10], *p* < 0.05) scores. MADRS scores were also significantly associated with Total AUDIT scores (B_2_ = 0.34, 95% CI [0.16, 0.52], *p* < 0.01), and a trend‐level association was shown between STAI and Total AUDIT scores (B_1_ = 0.14, 95% CI [−0.01, 0.31], *p* = 0.07). The direct effect of BIS scores on Total AUDIT scores was no longer significant in the presence of the mediators (direct effect = 0.11, *p* = 0.60). The indirect effect, estimated using a percentile bootstrap estimation, was significant as the CI did not contain 0 (indirect effect = 0.38, 95% CI [0.12, 0.66]). Approximately 77% of the relationship between BIS scores and Total AUDIT scores was explained by STAI and MADRS scores (indirect effect/total effect = 0.38/0.50).

**FIGURE 2:**
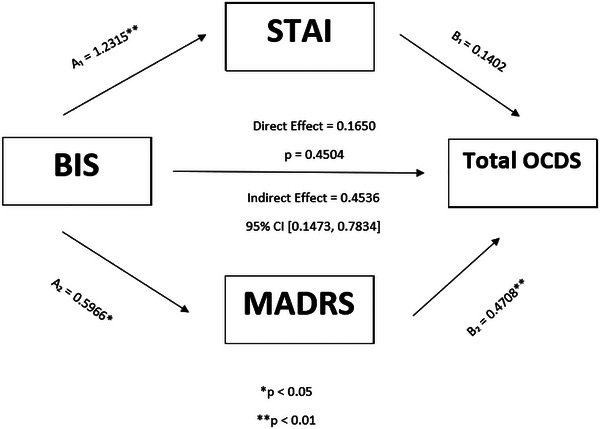
Parallel mediation with STAI and MADRS as mediators of the relationship between BIS and Total OCDS (controlling for age).

Figure 2 depicts the parallel mediation effects of STAI and MADRS scores on the relationship between BIS and Total OCDS scores. A significant indirect effect mediated by STAI and MADRS scores was found. Specifically, BIS scores were significantly associated with STAI (A_1_ = 1.23, 95% CI [0.67, 1.79], *p* < 0.01) and MADRS (A_2_ = 0.59, 95% CI [0.08, 1.10], *p* < 0.05) scores. MADRS scores were also significantly associated with Total OCDS scores (B_2_ = 0.47, 95% CI [0.29, 0.64], *p* < 0.01), and a trend‐level association was shown between STAI and Total OCDS scores (B_1_ = 0.14, 95% CI [−0.02, 0.30], *p* = 0.08). The direct effect of BIS scores on Total OCDS scores was no longer significant in the presence of the mediators (direct effect = 0.16, *p* = 0.45). The indirect effect, estimated using a percentile bootstrap estimation, was significant as the CI did not contain 0 (indirect effect = 0.45, 95% CI [0.12, 0.66]). Approximately 73% of the relationship between BIS scores and Total OCDS scores was explained by STAI and MADRS scores (indirect effect/total effect = 0.45/0.61).

Single mediation analyses were also conducted, and the results are presented in Appendix S9.

## 4 Discussion

This study investigated the relationship between the behavioral inhibition and approach systems and alcohol‐related outcomes in adult individuals with AUD. We examined the association between these two systems, as assessed by the BIS/BAS Scale, and severity of problematic alcohol use, levels of alcohol consumption, and alcohol‐related obsession and compulsion. Results showed that higher BIS scores were associated with higher obsessive and compulsive thoughts about alcohol, as measured by the OCDS, as well as with higher severity of problematic alcohol use, as measured by the AUDIT. Furthermore, post‐hoc analyses found that the observed associations between BIS scores and both OCDS and AUDIT scores were mediated by anxiety and depression levels, as measured by STAI and MADRS, respectively. No significant associations were found between BAS scores and alcohol‐related outcomes. Not fully confirming our original hypotheses, these results demonstrate a complex and probably non‐linear relationship between these two motivational systems and alcohol‐related outcomes that could be influenced by severity and length of alcohol use, stage of addiction, and mediating mental health factors, among others.

We found that BIS scores positively correlated with hazardous drinking (AUDIT‐H), but not with the other AUDIT subscales, consumption (AUDIT‐C) or dependence (AUDIT‐D). Hazardous drinking is generally characterized by an amount or pattern of alcohol use that considerably increases the risk of negative health outcomes (e.g., high blood pressure and liver disease) (Reid et al. 1999). Lack of a significant relationship between AUDIT‐C and BIS is consistent with our other results showing no association with alcohol consumption according to TLFB. Collectively, these results indicate that hazardous drinking (AUDIT‐H) is mainly driving the relationship between overall severity of alcohol use (Total AUDIT) and BIS scores, implying that BIS may not increase drinking in the low‐risk range, but potentially does once drinking reaches hazardous levels. However, this hypothesis should be further examined in a more heterogeneous sample with a wider range of alcohol use severity, as the current study included few participants with low levels of alcohol use. For example, as an AUD sample, only 2 out of the 94 participants did not meet criteria for heavy drinking (i.e., more than 7 or 14 drinks per week for females or males, respectively). Similar to our results, another study found a positive association between BIS and hazardous drinking in a community sample of adults (Hamilton et al. 2012). By contrast, other studies investigating this relationship in younger populations found no association (Franken et al. 2006; Loxton and Dawe 2001). A potential reason why some of the aforementioned studies differed from ours is that these studies primarily focused on younger individuals (typically college students) with shorter lifetime drinking history who typically have a lower prevalence and severity of AUD and follow a different drinking pattern (e.g., binge drinking) compared to our sample that included primarily middle‐aged individuals all of whom had a current diagnosis of AUD. Older adults with higher BIS scores who are in later stages of addiction may tend to use alcohol to cope with symptoms related to stress and anxiety (Koob et al. 2014), whereas younger people with higher BIS scores may avoid alcohol for its unfavorable outcomes. This notion implies that the BIS could impact alcohol‐related outcomes in individuals differently as they progress through the addiction cycle and develop different AUD‐related phenotypes, including more hazardous drinking patterns (Koob and Volkow 2010; Koob and Vendruscolo 2023). Other potential explanations for this association include increased drinking as a way to manage anxiety resulting from elevated punishment sensitivity due to higher BIS and/or a vicious cycle of hazardous drinking leading to increased anxiety about drinking and hence resulting in more hazardous drinking to cope with the anxiety (Hamilton et al. 2012).

Regarding the relationship between BIS scores and alcohol obsession and compulsivity, there seems to be a stronger positive association between BIS scores and compulsive drinking than obsessive drinking. In relation to alcohol, obsession is generally defined as preoccupation with alcohol, and compulsion is described as the behavioral and motivational aspects of alcohol consumption (Nakovics et al. 2008). Interestingly, a study investigating the relationship between the Iowa Gambling Task (IGT), a common measure of compulsivity, severity of alcohol drinking, and BIS found that participants with higher BIS sensitivity and higher alcohol consumption performed worse on the IGT, suggesting higher levels of compulsivity, whereas participants with higher BIS sensitivity and lower alcohol consumption performed better on the IGT (Logge et al. 2023). This is consistent with our study, since we studied participants with AUD and heavy drinking, and found that higher BIS sensitivity was associated with increased compulsive drinking. Overall, further work is needed to understand the link between the BIS and alcohol‐related outcomes, especially hazardous drinking and alcohol compulsion.

Originally, we hypothesized that higher BAS scores would be associated with more alcohol drinking, and higher BIS scores would be associated with less alcohol consumption. Because the BAS is thought to be activated by reward‐related cues, while the BIS is thought to be responsible for the defensive approach to dangerous or unpleasant situations (Vecchione et al. 2021), we hypothesized that individuals with a higher BAS score would be more likely to seek the rewarding effects of alcohol, resulting in more severe alcohol‐related outcomes. We found no significant relationship between BAS scores and any of the examined alcohol‐related outcomes in this sample. Previous studies suggest that higher BAS scores are associated with higher rates of problematic substance and alcohol use (Franken et al. 2006; Rieser et al. 2019; Wardell et al. 2011). However, in one study that compared alcohol‐dependent people after 8 weeks of abstinence with controls, higher BAS scores were associated with increased length of alcohol abstinence (Sprah and Novak 2008), implying that the link is not straightforward and may depend on the length and stage of addiction, among other factors. Of note, that study was conducted only in males and had a small sample size (Sprah and Novak 2008). One possible explanation for the lack of association between BAS and alcohol‐related outcomes in our study may be related to specific aspects of motivation for consuming alcohol in this sample of middle‐aged heavy‐drinking individuals with relatively severe AUD. Rather than consuming alcohol for positive reinforcement and rewarding effects, such as feelings of euphoria and disinhibition, participants in our study were probably more likely to seek alcohol for negative reinforcement and to cope with stress and negative emotional/motivational symptoms (Studer et al. 2016; Koob 2022).

We also hypothesized that individuals with higher BIS scores may worry more about the harmful impact of alcohol and avoid consumption, resulting in less severe alcohol‐related outcomes. However, we found the opposite, in this sample, in the relationship between BIS scores and severity of alcohol use (AUDIT) and alcohol obsession and compulsivity (OCDS). Previous research has found mixed results on the link between the BIS and alcohol use, with some studies finding an inverse relationship between BIS score and alcohol use (Kimbrel et al. 2007; O'Connor et al. 2009; Pardo et al. 2007), one finding a positive correlation (Voigt et al. 2009), and others finding no associations (Hundt et al. 2008; Knyazev 2004). One potential source of inconsistency in these results is that while the BIS is typically associated with anxiety, negative affect, and coping‐related drinking (Keough and O'Connor 2014), it could also protect against problematic drinking because alcohol can have undesirable outcomes, e.g., a lost sense of control (Corr 2004). Unlike most of the previous studies that included college students and younger individuals, ours included an older sample of heavy‐drinking adults with AUD and a much longer lifetime drinking history. College students tend to drink alcohol in a binge‐like pattern and primarily for its rewarding properties, while older people with more advanced AUD tend to drink alcohol to reduce stress, negative emotional states, and withdrawal symptoms. Three stages of addiction have been proposed: binge/intoxication, withdrawal/negative affect, and preoccupation/anticipation, with changes in motivation being a key component of developing an AUD (Koob and Volkow 2010; Koob 2022). Early stages of addiction are often dominated by positive reinforcement, while later and more advanced stages are primarily driven by negative reinforcement and automaticity, although it is also understood that people could enter the addiction cycle at any of these stages (Koob and Volkow 2010). College students who engage in binge drinking are more likely to be in earlier stages of the addiction cycle, where they are motivated by the rewarding properties of alcohol, whereas our heavy‐drinking participants with AUD are more likely to be driven by negative reinforcement. This notion implies that the BIS could impact alcohol‐related outcomes in individuals differently during their lifespan and as they progress through different stages of alcohol use and AUD—an interpretation that is speculative at this point. Fully factorial and longitudinal studies assessing the relationship between the BIS and alcohol‐related outcomes across different groups and over time are needed to test this hypothesis.

In addition to the well‐established link between anxiety/depression and AUD, prior research has found a relationship between the BIS and anxiety symptoms (Corr 2004; Bijttebier et al. 2009; Torvik et al. 2019; Gimeno et al. 2017), as well as a relationship between BIS and depressive symptoms (Li et al. 2015; Kasch et al. 2002; Boden and Fergusson 2011). Accordingly, we speculated that anxiety‐ and/or depression‐related symptoms may mediate the relationship between BIS and alcohol‐related outcomes. Presumably, higher BIS would predict higher anxiety/depression, and higher anxiety/depression would predict higher alcohol craving and use. The results of our post‐hoc mediation analysis confirmed this hypothesis and showed significant mediation by anxiety (STAI) and depression (MADRS) in the relationships between BIS and severity of alcohol use (AUDIT) and alcohol obsession and compulsion (OCDS). Our findings suggest that the observed positive association between BIS scores and these alcohol‐related outcomes can be considerably explained by anxiety and depression symptoms. These results are consistent with a previous study that found anxiety to be a contributing factor when higher BIS is associated with more severe alcohol use (Keough and O'Connor 2014). Collectively, our mediation analyses suggest that: (A) Anxiety and depression symptoms explain, at least in part, the association between BIS and alcohol use, especially in the context of hazardous alcohol use and AUD, and (B) varying levels of anxiety and depression (and possibly other unmeasured factors) in study samples may explain some the inconsistencies in previous findings on the link between BIS/BAS and alcohol‐related outcomes. Here, we used two validated scales that measure levels of anxiety (STAI) and depression (MADRS), and the availability of data across the spectrum enabled us to conduct mediation analyses. These assessments quantify symptoms but do not equate to DSM diagnoses of anxiety or depressive disorders (Shah et al. 2021; Hobden et al. 2017)—an additional aspect that should be explored in larger future studies. We also explored BAS variables and treatment‐seeking status as possible moderators of the link between BIS and alcohol‐related outcomes, none of which showed significant effects in the context of this study and in this sample.

This study has several limitations. The sample was relatively small and predominantly composed of middle‐aged males, which may limit the generalizability of the findings. Of note, due to restrictive inclusion and exclusion criteria in some of the parent studies (related to the interventions tested in the parent studies and unrelated to the present study), only 20 females were included, making it difficult to extend the results to the general female population or examine possible sex differences. Future research should include more diverse and representative samples, including a more balanced sample across sexes. Relatedly, a strength of this study is its racial heterogeneity; for example, 50% of the sample identified as Black/African American—a population often underrepresented in this area of research. Race was tested as a covariate in all regression models and did not come out statistically significant, but we did not specifically examine race differences due to the small number of each subgroup—a question that can be addressed in larger studies. An important characteristic of this study was that all participants had a current diagnosis of AUD, and all but two participants met criteria for heavy drinking. While this is a novel and clinically relevant aspect, it also poses limitations, e.g., inability to examine potential differences across drinking groups and levels (e.g., people with vs. without AUD, heavy‐ vs. non‐heavy‐drinkers). That said, this rich secondary dataset with deep phenotyping enabled us to analyze several alcohol‐related outcomes and possible mediators/moderators in relation to BIS/BAS, generating hypotheses to be tested in future larger and fully powered studies. Our study used solely self‐report questionnaires, which could result in recall bias or under‐/over‐reporting. As previously described, this work is a secondary analysis of data pooled and aggregated from several alcohol‐related studies that were not a priori designed towards the specific questions asked here. This limitation is somewhat offset by the fact that all data analyzed were collected at baseline and prior to the initiation of any study procedures or administration of study medications. Our study cannot establish causality given its cross‐sectional design. Although meaningful associations were identified, longitudinal research is needed to clarify the temporal link between different facets of BIS/BAS and alcohol‐related outcomes, especially in people with AUD.

In conclusion, our findings suggest that a more active BIS in heavy‐drinking individuals with AUD is associated with more severe AUD and more obsession and compulsion towards alcohol, and that anxiety and depression symptoms partially mediate these relationships. Additional prospective studies with larger and more representative samples are necessary to gain a deeper insight into the relationship between BIS/BAS and alcohol‐related outcomes.

## Author Contributions

**Madeline E. Crozier**: conceptualization, methodology, formal analysis, investigation, data curation, writing–original draft, visualization. **Lorenzo Leggio**: conceptualization, investigation, resources, writing–review and editing, supervision, funding acquisition; **Mehdi Farokhnia**: conceptualization, methodology, formal analysis, investigation, data curation, writing–review and editing, supervision, project administration.

## Disclosure

This research was supported by the Intramural Research Program of the National Institutes of Health (NIH). The contributions of the authors are considered Works of the United States Government. The findings and conclusions presented in this paper are those of the authors and do not necessarily reflect the views of the NIH or the U.S. Department of Health and Human Services.

## Peer Review

The peer review history for this article is available at https://publons.com/publon/10.1002/brb3.70944.

## Supporting information




**Supporting Materials**: brb370944‐sup‐0001‐SuppMatt.docx

## Data Availability

The data that support the findings of this study are available from the corresponding author upon reasonable request.
