# Supplementary material for: Exploring the Relationship Between Behavioral Inhibition and Approach Systems and Alcohol‐Related Outcomes in People With Alcohol Use Disorder
Source: Brain Behav. 2025 Oct 20;15(10):e70944. doi: 10.1002/brb3.70944 (PMC12537829; doi:10.1002/brb3.70944)

**Supplementary Information**

**Table of Contents:**

- **Appendix S1.** Eligibility Criteria of Study 1 (NCT01751386)
- **Appendix S2.** Eligibility Criteria of Study 2 (NCT01779024)
- **Appendix S3.** Eligibility Criteria of Study 3 (NCT02039349)
- **Appendix S4.** Eligibility Criteria of Study 4 (NCT02707055)
- **Appendix S5.** Eligibility Criteria of Study 5 (NCT03152760)
- **Figure S1:** Flow Chart
- **Appendix S6.** Full Bivariate Correlation Table
- **Appendix S7.** Full Regression Tables
- ***Table S1.*** *Alcohol Use Disorder Identification Test (AUDIT) score as outcome without controlling for demographics*
- ***Table S2.*** *AUDIT score as outcome with controlling for demographics*
- ***Table S3.*** *Obsessive-Compulsive Drinking Scale (OCDS) score as outcome without controlling for demographics*
- ***Table S4.*** *OCDS score as outcome with controlling for demographics*
- **Appendix S8:** Mediating Variables (STAI and MADRS) Regression Tables
- ***Table S5.*** *Spielberger State-Trait Anxiety Inventory (STAI) score with controlling for demographics*
- ***Table S6.*** *Montgomery-Asberg Depression Rating Scale (MADRS) score with controlling for demographics*
- **Appendix S9.** Single Mediation Analyses
- ***Figure S2.*** *Single mediation with STAI as a mediator between BIS and Total AUDIT (controlling for age)*
- ***Figure S3.*** *Single mediation with MADRS as a mediator between BIS and Total AUDIT*
- ***Figure S4.*** *Single mediation with STAI as a mediator between BIS and Total OCDS (controlling for age)*
- ***Figure S5.*** *Single mediation with MADRS as a mediator between BIS and Total OCDS*

**List of Abbreviations:**

| ALT | Alanine Transaminase |
| --- | --- |
| AST | Aspartate Transferase |
| AUD | Alcohol Use Disorder |
| AUDIT | Alcohol Use Disorders Identification Test |
| BAS | Behavioral Approach System |
| BIS | Behavioral Inhibition System |
| BMI | Body Mass Index |
| CIWA-Ar | Clinical Institute Withdrawal Assessment for Alcohol - Revised |
| CNS | Central Nervous System |
| DPP4 | Dipeptidyl Peptidase-4 |
| DSM | Diagnostic and Statistical Manual of Mental Disorders |
| ECG | Electrocardiogram |
| fMRI | Functional Magnetic Resonance Imaging |
| FSH | Follicle-Stimulating Hormone |
| hCG | Human Chorionic Gonadotropin |
| IM | Intramuscular |
| IUD | Intrauterine Device |
| IV | Intravenous |
| MADRS | Montgomery-Asberg Depression Rating Scale |
| MAI | Medical Advisory Investigator |
| MDD | Major Depressive Disorder |
| MRI | Magnetic Resonance Imaging |
| NIAAA | National Institute on Alcohol Abuse and Alcoholism |
| OCDS | Obsessive-Compulsive Drinking Scale |
| OTC | Over the Counter |
| PRN | Pro Re Nata |
| QTc | QT interval corrected for standard heart rate of 60 beats per minute |
| SCID | Structural Clinical Interview for DSM Disorders |
| STAI | Spielberger State-Trait Anxiety Inventory |
| TLFB | Timeline Follow-Back |
| TZD | Thiazolidinediones |

**Appendix S1.** Eligibility Criteria of Study 1 (NCT01751386)

*Inclusion Criteria:*

1. Male or female between 21 and 65 years old (inclusive)
2. Current DSM-IV-TR diagnosis of alcohol dependence, supported by the Structural Clinical Interview for DSM-IV-TR Axis I Disorders (SCID)
3. A Trait STAI score>39
4. Good health as confirmed by medical history, physical examination, ECG, blood/urine lab tests
5. Females: postmenopausal for at least one year, surgically sterile, or practicing an effective method of birth control before entry and throughout the study; negative urine pregnancy test at each visit. Reliable methods of birth control include oral contraceptives or Norplant®; barrier methods such as diaphragms with contraceptive jelly, cervical caps with contraceptive jelly, condoms with contraceptive foam, or intrauterine devices; a partner with a vasectomy; or abstinence from intercourse.

*Exclusion Criteria:*

1. Expressed interest in treatment for alcoholism and/or anxiety
2. Females: pregnant, breast-feeding, or not using an adequate form of birth control
3. Unable to provide a negative urine drug screen
4. Diagnosis of current substance dependence, other than alcohol or nicotine
5. Lifetime diagnosis of schizophrenia, bipolar disorder, or other psychoses
6. Active illness within 6 months prior to Visit 1 that meets DSM-IV criteria for diagnosis of Major Depressive Disorder (MDD), or a history of attempted suicide
7. Clinically significant medical abnormalities (i.e., unstable hypertension, clinically significant ECG abnormalities, Creatinine>2 mg/dL) or clinically significant liver problems (i.e. liver cirrhosis, AST or ALT>5x the upper normal limit, Hepatitis B and C)
8. Current use of psychotropic medications that cannot be discontinued and that may have an effect on alcohol consumption or that may interact with baclofen (specifically including: naltrexone, acamprosate, alcohol dehydrogenase inhibitors, topiramate, gabapentin, ondansetron, benzodiazepines, beta-blockers, H2-blockers, and alpha-1 blockers)
9. Medical contraindications for use of baclofen
10. A history of adverse reaction or hypersensitivity to baclofen
11. Significant alcohol withdrawal symptoms, defined as a CIWA-Ar > 8
12. History of epilepsy or alcohol-related seizures
13. Poor venous access based on physical exam and medical history

**Appendix S2.** Eligibility Criteria of Study 2 (NCT01779024)

*Inclusion criteria*

1. Male and female participants between 21-60 years of age
2. Good health as determined by medical history, physical exam, ECG, and lab tests
3. Creatinine ≤ 2 mg/dl
4. Female must have a negative urine pregnancy (hCG) test at the start of each study session. Females of childbearing potential who are sexually active and have not been surgically sterilized must agree to use an adequate method of birth control during the study. Adequate methods of contraception for sexually active women are having a male sexual partner(s) who is surgically sterilized prior to inclusion; having a sexual partner(s) who is/are exclusively female; using oral contraceptives (either combined or progestrogen only) with a single-barrier method of contraception consisting of spermicide and condom or diaphragm; using double-barrier contraception, specifically, a condom plus spermicide and a female diaphragm or cervical cap plus spermicide; or using an approved intrauterine device (IUD) with established efficacy
5. Participants must drink alcohol regularly at a heavy level, on average greater than 20 drinks per week for men, and greater than 15 drinks per week for women, and not be seeking help for alcohol-related problems
6. Participant must be willing to receive two IV lines

*Exclusion criteria*

1. Current or prior history of any clinically significant disease, including CNS, cardiovascular, respiratory, gastrointestinal, hepatic, renal, endocrine, or reproductive disorders
2. Specific exclusion criteria related to the administration of ghrelin, are chronic inflammatory diseases (e.g., Crohn’s disease, ulcerative colitis, celiac disease), diabetes, obesity (BMI ≥ 30 kg/m^2^), weight ≥ 120 Kg, high triglycerides level (> 350 mg/dL), history of clinically significant hypotension (e.g.: history of fainting and/or syncopal attacks) and/or resting systolic BP < 100 mmHg
3. Positive hepatitis or HIV test at screening
4. Current clinically significant major depression or anxiety; or prior clinically significant psychiatric problems, including eating disorders, schizophrenia, bipolar disorder, obsessive compulsive disorder
5. Current diagnosis of substance dependence (other than alcohol or nicotine)
6. Currently seeking treatment for alcohol use disorder
7. History of significant withdrawal symptoms or presence of clinically significant withdrawal symptoms (Clinical Institute Withdrawal Assessment (CIWA) score > 8) at screening
8. Non-drinkers (alcohol-naïve individuals or current abstainers) or no experience drinking 5 or more drinks on one occasion
9. Unable to provide a negative urine drug screen
10. Pregnancy or intention to become pregnant for women. Female participants will undergo a urine beta-hCG test to ensure they are not pregnant
11. Use of prescription or OTC medications known to interact with alcohol within 2 weeks of the study. These include, but may not be limited to: isosorbide, nitroglycerine, benzodiazepines, warfarin, anti-depressants such as amitriptyline, clomipramine and nefazodone, anti-diabetes medications such as glyburide, metformin and tolbutamide, H2-antagonists for heartburn such as cimetidine and ranitidine, muscle relaxants, anti-epileptics including phenytoin and phenobarbital codeine, and narcotics including darvocet, ercocet and hydrocodone. Drugs known to inhibit or induce enzymes that metabolize alcohol should not be used for 4 weeks prior to the study. These include chlorzoxazone, isoniazid, metronidazole and disulfiram. Cough-and-cold preparations, which contain antihistamines, pain medicines and anti-inflammatories such as aspirin, ibuprofen, acetaminophen, celecoxib and naproxen, should be withheld for at least 72 hours prior to each study session
12. Current or prior history of alcohol-induced flushing reactions
13. Contraindications for MRI scanning, including metal in body that are contraindicated for MRI (such as implants, pacemaker, prostheses, shrapnel, irremovable piercings), left-handedness, and claustrophobia

**Appendix S3.** Eligibility Criteria of Study 3 (NCT02039349)

*Inclusion criteria*

1. Males or females 21-65 years old (inclusive)
2. Heavy drinking defined as on average at least 21 drinks per week for men or at least 14 drinks for women based on the timeline follow-back (TLFB) done at screening
3. Be in good health as confirmed by medical history, physical examination, ECG, blood/urine lab tests

4. Female subjects must be of non‑childbearing potential as defined by at least one of the following criteria:

a) Females 45 – 65 years old, who are menopausal, defined as follow:

i) Females who are between 45 – 55 years old: they will be considered menopausal if they satisfy all the following three requirements during screening: 1) they are in amenorrhea, defined as absence of menstruation for the previous 12 months; 2) they have a negative urine pregnancy test; and 3) they have a serum FSH level within the laboratory’s reference range for postmenopausal females.

ii) Females who are between 56 – 65 years old: they will be considered menopausal if they are in amenorrhea, defined as absence of menstruation for the previous 12 months before screening.

**OR**

b) Females 21-65 years old, who have a documented hysterectomy and/or bilateral oophorectomy.

All other female subjects (including females with tubal ligations and females that do NOT have a documented hysterectomy) will be considered to be of childbearing potential.

1. Male subjects must use one of the following methods of contraception from the first dose of study medication and until 28 days after dosing:
2. Abstinence
3. A condom AND one of the following:

- Vasectomy for more than 6 months.
- Female partner who meets one of the following conditions:

1. Has had a tubal ligation, hysterectomy, or bilateral oophorectomy
2. Is post‑menopausal
3. Uses one of the following forms of contraception:

- Copper or hormonal containing IUD
- Spermicidal foam/gel/film/cream/suppository
- Diaphragm with spermicide
- Oral contraceptive
- Injectable progesterone
- Subdermal implant

*Exclusion criteria*

1. Interest in receiving treatment for heavy drinking
2. Current DSM-IV diagnosis (based on SCID) of substance dependence (other than alcohol and/or nicotine); a negative urine drug screen will also be required
3. DSM-IV Axis I criteria for a lifetime diagnosis of schizophrenia, bipolar disorder, or other psychoses
4. Active illness within the past 6 months of the screening visit that meet the DSM-IV criteria for a diagnosis of major depressive disorder or anxiety disorder; subjects with a history of attempted suicide will be excluded
5. Clinically significant medical abnormalities (e.g., unstable hypertension, clinically significant EKG abnormalities, Creatinine ≥ 2 mg/dL, liver cirrhosis, AST or ALT > 3x the upper normal limit, hemoglobin <10.5 g/dl)
6. Heart rate >100 at screening on two separate measurements given potential of study medication to increase heart rate
7. BMI ≤ 18.5 or anorexia given potential of the study medication to reduce appetite
8. BMI ≥ 35 kg/m^2^
9. Exclusionary Medications:
   1. Naltrexone, acamprosate, alcohol dehydrogenase inhibitors, topiramate, gabapentin, ondansetron, benzodiazepines, beta-blockers, H2-blockers, and alpha-1 blockers, baclofen, and barbiturates as well as hormone replacement therapy; medications and dietary/herbal supplements (like St. John’s wort) that interact with Cytochrome P450 3A4. All of the medications in the previous sentence will not be allowed if they have been taken within 2 weeks of study medication administration.
   2. PF-05190457 is a substrate for P-glycoproteins (P-gp or encoded by ABCB1 gene) based on information from in vitro or animal models. Patients that are required to take the following inhibitors and inducers of P-gp are excluded unless the subject stops taking these agents for 2 weeks for P-gp inhibitors or 6 weeks for P-gp inducers before study medication administration.

Inhibitors: Amiodarone, azithromycin, captopril, carvedilol, clarithromycin, conivaptan, cyclosporine, diltiazem, dronedarone, erythromycin, felodipine, itraconazole, ketoconazole, lopinavir and ritonavir, quercetin, quinidine, ranolazine, verapamil

Inducers: Avasimibe, carbamazepine, phenytoin, rifampin, St John’s wort, tipranavir/ritonavir

1. History of epilepsy or alcohol-related seizures
2. patients who have diabetes and/or are treated with any drug with glucose lowering properties such as sulfonylurea, insulin, metformin, thiazolidinediones (TZD), Dipeptidyl peptidase-4(DPP4) inhibitors, or Glucagon-like peptide-1 (GLP-1) agonists (due to the glucose-lowering properties of PF-05190457 observed in healthy volunteers)
3. History of alcohol-induced flushing reactions
4. Clinically significant alcohol withdrawal symptoms, as assessed by a CIWA-Ar score > 8 at screening
5. Any other reason or clinical condition for which the PI or the MAI will consider unsafe for a possible participant to participate in this study

**Appendix S4.** Eligibility Criteria of Study 4 (NCT02707055)

*Inclusion criteria*

1. Male or female individuals 18-70 years old (inclusive)
2. Current Alcohol Use Disorder (AUD) by DSM-5 criteria based on the SCID
3. Most recent urine drug test for illegal drugs of abuse is negative
4. Most recent Clinical Institute Withdrawal Assessment for Alcohol – revised (CIWA-Ar) score is ≤ 8
5. Heart rate ≤ 100 on two separate measurements, both assessed after CIWA-Ar score is ≤ 8
6. Female subjects must be of non‑childbearing potential as defined by at least one of the following criteria:

a) Females 45 –70 years old, who are menopausal, defined as follow:

i) Females who are between 45 – 55 years old: they will be considered menopausal if they satisfy all the following three requirements during screening: 1) they are in amenorrhea, defined as absence of menstruation for the previous 12 months; 2) they have a negative urine pregnancy test; and 3) they have a serum FSH level within the laboratory’s reference range for postmenopausal females.

ii) Females who are between 56 – 70 years old: they will be considered menopausal if they are in amenorrhea, defined as absence of menstruation for the previous 12 months before screening.

OR

1. Females 21-70 years old, who have a documented hysterectomy and/or bilateral oophorectomy.

**NOTE:** All other female subjects (including females with tubal ligations and females that do NOT have a documented hysterectomy) will be considered of childbearing potential.

1. Male subjects must use one of the following methods of contraception from the first dose of study medication and until 28 days after dosing (given that it is unknown whether the effects of this drug can cause birth defects):
2. Abstinence.
3. A condom AND one of the following:
4. Vasectomy for more than 6 months
5. Female partner who meets one of the following conditions:
6. Has had a tubal ligation, hysterectomy, or bilateral oophorectomy
7. Is post‑menopausal
8. Uses one of the following forms of contraception:

- Copper or hormonal containing IUD
- Spermicidal foam/gel/film/cream/suppository
- Diaphragm with spermicide
- Oral contraceptive
- Injectable progesterone
- Subdermal implant

*Exclusion criteria*

1. Lifetime clinical diagnosis of schizophrenia or bipolar disorder
2. EKG with QTc > 450 msec as determined by the Fridericia formula
3. BMI < 18.5 kg/m^2^ or anorexia
4. BMI ≥ 40 kg/m^2^
5. History of epilepsy and/or seizures

**NOTE:** individuals who have a history of alcohol withdrawal seizures may be in the study as long as they have been abstinent from alcohol for at least 2 weeks prior to consent and during that period of abstinence, there were no seizure episodes (otherwise, participant remains not eligible).

1. Most recent blood tests show creatinine ≥ 2 mg/dL, AST or ALT > 3x the upper normal limit, hemoglobin <10.5 g/dl
2. Subjects who have diabetes and/or are treated with any drug with glucose lowering properties such as sulfonylurea, insulin, metformin, thiazolidinediones (TZD), Dipeptidyl peptidase-4 (DPP4) inhibitors, or Glucagon-like peptide-1(GLP-1) agonists (due to the glucose-lowering properties of PF-05190457 observed in healthy volunteers)
3. Exclusionary Medications: Naltrexone, acamprosate, alcohol dehydrogenase inhibitors, topiramate, gabapentin, ondansetron, benzodiazepines, and alpha-1 blockers, baclofen, drugs that are known to prolong the QTc interval and barbiturates as well as hormone replacement therapy; medications and dietary/herbal supplements (like St. John’s wort) that interact with Cytochrome P450 3A4. Patients who take these medications may be enrolled in the study only if the potentially interacting medication has been stopped for a period of at least 5 half-lives of the interacting medication before PF-05190457 administration. Patients who take these medications on an as needed (PRN) schedule or take the medication as a one-time dose as part of a medical procedure or a diagnostic test, for example, may not have to wait the 5 half-lives’ period of time before enrollment; this will be evaluated on a case-by-case basis by the MAI and/or PI, based on the specific pharmacological properties of the medication.
4. Unable to pass a finger rub hearing test
5. Vision is unable to be corrected to (Snellen) 20/100
6. Clinically significant history of motion or car sickness, or history of vestibular disorders
7. Any other reason or clinical condition for which the PI or the MAI will consider unsafe for a possible participant to participate in this study

*Exclusion Criteria for fMRI only****:***

1. Have contraindications for brain fMRI, as determined by the NIAAA MRI Safety screening form (conducted under the 14-AA-0181 Screening Protocol)
2. Colorblindness (this would prevent subject from completing the Stroop task) using the Ishihara Test for Color Deficiency, Concise Edition, 2014

**Appendix S5.** Eligibility Criteria of Study 5* (NCT03152760)

*Inclusion Criteria:*

*All Participants*

- - - 1. Male or female individuals 21-70 years old (inclusive)

*Specific for Abstinent Group: AB*

- 1. Current Alcohol Use Disorder (AUD) by DSM-5 criteria
  2. Being alcohol abstinent for at least 4 weeks, with a minimum of 2 weeks in a non-protective environment at the time of study screening

*Specific for Current Drinking Group: CD*

- - 1. Current AUD by DSM-5 criteria
    2. Participants not seeking treatment for their alcohol use will be included
  1. Satisfying heavy drinking criteria during the 4-weeks prior to screening (“for men, >14 standard drinks in any one week and ≥4 drinks per occasion at least once per month over the past 30 days; for women, >7 drinks per week and ≥3 drinks per occasion at least once per month over the past 30 days”) and any drinking during the 2-day prior to signing the study-specific consent

*Exclusion Criteria:*

*All Participants*

1. Current pregnancy or lactation
2. Positive Urine Drug Test for illegal drugs
3. Body Mass Index (BMI) < 18.5 kg/m2 or BMI ≥ 40 kg/m2
4. Presence of active implantable electronic devices (e.g., defibrillators, pumps, pacemakers)
5. The following current medical conditions: diabetes; chronic gut inflammatory diseases; GI or any other type of cancer; short bowel syndrome; conditions requiring parenteral nutrition
6. Diarrhea or other symptoms of possible enteritis in the past 7 days (self-reported)
7. Recent history of sigmoidoscopy or colonoscopy (past 30 days)
8. Current use (past 90 days) of the following medications: oral and/or IV antimicrobials (specifically: antiviral, antifungal, or antibiotics); prebiotics; probiotics; laxatives; antispasmodic drugs; oral, IM or IV steroids
9. Any other reason or clinical condition that the PI, or Medical Advisory Investigator (MAI) considers unsafe or not in the best interest of the study research integrity

**This study also included a healthy control (HC) control which is not included in the present analyses.*

**Figure S1:** Flow Chart

**N = 147***

Participants without AUD diagnosis were removed (n = 14)

**N = 133**

Participants without BIS/BAS data were removed (n = 6)

**N = 127**

Participants without AUDIT data were removed (n = 7)

**N = 120**

Participants without OCDS data were removed (n = 12)

**N = 108**

Repeated participants were removed (n = 12)

**N = 96**

Participants with outlying TLFB data (total drinks > 3500 drinks in 90 days) were removed (n = 2)

**N = 94**

**Total number of participants enrolled in the five parent studies*

**Appendix S6.** Full Bivariate Correlations Table

|  | **TLFB Average Drinks Per Drinking Day** | **TLFB Heavy Drinking Days** | **AUDIT C Score** | **AUDIT H Score** | **AUDIT D Score** | **Total AUDIT Score** | **Obsessive Drinking Score** | **Compulsive Drinking Score** | **Total OCDS Score** |
| --- | --- | --- | --- | --- | --- | --- | --- | --- | --- |
| **BIS Score** | | | | | | | | | |
| Spearman Correlation Coefficient (ρ) | -0.127 | -0.044 | -0.036 | .339 | 0.035 | 0.179 | 0.186 | .288 | 0.25 |
| P value | 0.222 | 0.676 | 0.729 | <0.001 | 0.735 | 0.085 | 0.073 | 0.005 | 0.015 |
| **BAS Drive Score** | | | | | | | | | |
| Spearman Correlation Coefficient (ρ) | -0.072 | -0.045 | -0.014 | 0.047 | 0.083 | 0.058 | 0.126 | 0.101 | 0.112 |
| P value | 0.493 | 0.667 | 0.891 | 0.654 | 0.426 | 0.577 | 0.226 | 0.333 | 0.282 |
| **BAS Fun-Seeking Score** | | | | | | | | | |
| Spearman Correlation Coefficient (ρ) | -0.049 | -0.049 | -0.028 | 0.025 | -0.005 | 0.012 | 0.051 | 0.115 | 0.071 |
| P value | 0.640 | 0.638 | 0.786 | 0.813 | 0.962 | 0.909 | 0.623 | 0.271 | 0.497 |
| **BAS Reward-Responsiveness Score** | | | | | | | | | |
| Spearman Correlation Coefficient (ρ) | 0.002 | 0.007 | -0.086 | 0.047 | -0.024 | 0.002 | -0.156 | -0.068 | -0.124 |
| P value | 0.987 | 0.946 | 0.411 | 0.651 | 0.816 | 0.984 | 0.133 | 0.514 | 0.234 |
| **BAS Average Score** | | | | | | | | | |
| Spearman Correlation Coefficient (ρ) | -0.033 | 0.000 | -0.022 | 0.093 | 0.047 | 0.068 | 0.042 | 0.103 | 0.064 |
| P value | 0.753 | 0.997 | 0.832 | 0.373 | 0.653 | 0.518 | 0.687 | 0.322 | 0.541 |

**Appendix S7:** Full Regression Tables

***Table S1.*** *AUDIT score as outcome without controlling for demographics*

| **Variable** | **B** | **Standard Error** | **β** | **t** | **p** |
| --- | --- | --- | --- | --- | --- |
| (Constant) | 14.849 | 4.262 |  | 3.484 | <0.001 |
| BIS score | 0.484 | 0.222 | 0.222 | 2.180 | 0.032 |
| R^2^ = 0.049 | | | | | |

***Table S2.*** *AUDIT score as outcome with controlling for demographics*

| **Variable** | **B** | **Standard Error** | **β** | **t** | **p** | **VIF** |
| --- | --- | --- | --- | --- | --- | --- |
| (Constant) | 23.354 | 7.140 |  | 3.271 | 0.002 |  |
| BIS score | 0.590 | 0.242 | 0.270 | 2.433 | 0.017 | 1.199 |
| Age | -0.072 | 0.074 | -0.103 | -0.973 | 0.333 | 1.084 |
| Race | 0.834 | 1.142 | 0.078 | 0.730 | 0.467 | 1.108 |
| Sex | 0.927 | 1.929 | 0.050 | 0.480 | 0.632 | 1.063 |
| Years of Education | -0.512 | 0.220 | -0.259 | -2.330 | 0.022 | 1.201 |
| BMI | -0.120 | 0.182 | -0.070 | -0.659 | 0.512 | 1.088 |
| Smoking Status | 0.156 | 1.703 | 0.010 | 0.092 | 0.927 | 1.181 |
| R^2^ = 0.117 | | | | | | |

***Table S3.*** *OCDS score as outcome without controlling for demographics*

| **Variable** | **B** | **Standard Error** | **β** | **t** | **p** |
| --- | --- | --- | --- | --- | --- |
| (Constant) | 7.683 | 4.506 |  | 1.705 | 0.092 |
| BIS score | 0.544 | 0.235 | 0.235 | 2.318 | 0.023 |
| R^2^ = 0.055 | | | | | |

***Table S4.*** *OCDS score as outcome with controlling for demographics*

| **Variable** | **B** | **Standard Error** | **β** | **t** | **p** | **VIF** |
| --- | --- | --- | --- | --- | --- | --- |
| (Constant) | 21.045 | 7.538 |  | 2.792 | 0.006 |  |
| BIS score | 0.769 | 0.256 | 0.332 | 3.006 | 0.003 | 1.199 |
| Race | -0.126 | 0.078 | -0.169 | -1.611 | 0.111 | 1.084 |
| Sex | -0.445 | 1.206 | -0.039 | -0.369 | 0.713 | 1.108 |
| Age | 0.615 | 2.037 | 0.031 | 0.302 | 0.763 | 1.063 |
| Years of Education | -0.335 | 0.232 | -0.160 | -1.443 | 0.153 | 1.201 |
| BMI | -0.221 | 0.193 | -0.121 | -1.149 | 0.254 | 1.088 |
| Smoking Status | -1.126 | 1.798 | -0.069 | -0.626 | 0.533 | 1.181 |
| R^2^ = 0.125 | | | | | | |

**Appendix S8:** Mediating Variables (STAI and MADRS) Regression Tables

***Table S5.*** *STAI score as outcome with controlling for demographics*

| **Variable** | **B** | **Standard Error** | **β** | **t** | **p** | **VIF** |
| --- | --- | --- | --- | --- | --- | --- |
| (Constant) | 37.423 | 8.957 |  | 4.178 | <0.001 |  |
| BIS score | 1.354 | 0.304 | 0.464 | 4.455 | <0.001 | 1.199 |
| Age | -0.216 | 0.093 | -0.231 | -2.331 | 0.022 | 1.084 |
| Race | -0.204 | 1.433 | -0.014 | -0.143 | 0.887 | 1.108 |
| Sex | -1.824 | 2.420 | -0.074 | -0.754 | 0.453 | 1.063 |
| Years of Education | -0.417 | 0.276 | -0.158 | -1.511 | 0.134 | 1.201 |
| BMI | -0.071 | 0.229 | -0.031 | -0.312 | 0.756 | 1.088 |
| Smoking Status | 1.409 | 2.137 | 0.068 | 0.659 | 0.511 | 1.181 |
| R^2^ = 0.222 | | | | | | |

***Table S6.*** *MADRS score as outcome with controlling for demographics*

| **Variable** | **B** | **Standard Error** | **β** | **t** | **p** | **VIF** |
| --- | --- | --- | --- | --- | --- | --- |
| (Constant) | -1.844 | 8.078 |  | -0.228 | 0.820 |  |
| BIS score | 0.576 | 0.274 | 0.235 | 2.100 | 0.039 | 1.199 |
| Age | 0.035 | 0.084 | 0.044 | 0.413 | 0.681 | 1.084 |
| Race | 1.872 | 1.294 | 0.156 | 1.446 | 0.152 | 1.108 |
| Sex | 0.545 | 2.181 | 0.026 | 0.250 | 0.803 | 1.063 |
| Years of Education | -0.197 | 0.249 | -0.089 | -0.791 | 0.431 | 1.201 |
| BMI | 0.014 | 0.206 | 0.008 | 0.070 | 0.944 | 1.088 |
| Smoking Status | -1.867 | 1.926 | -0.107 | -0.969 | 0.335 | 1.181 |
| R^2^ = 0.110 | | | | | | |

**Appendix S9.** Single Mediation Analyses

***Figure S2.*** *Single mediation with STAI as a mediator between BIS and Total AUDIT (controlling for age)*


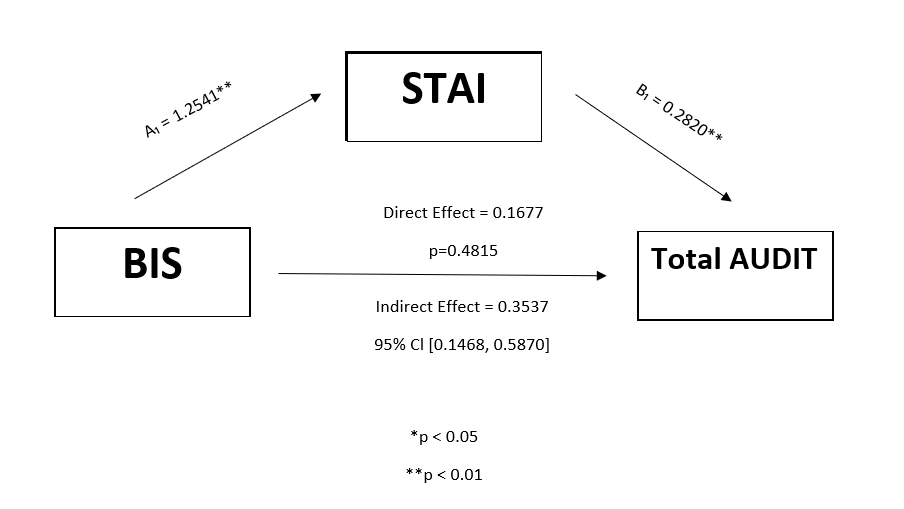


***Figure S3.*** *Single mediation with MADRS as a mediator between BIS and Total AUDIT*


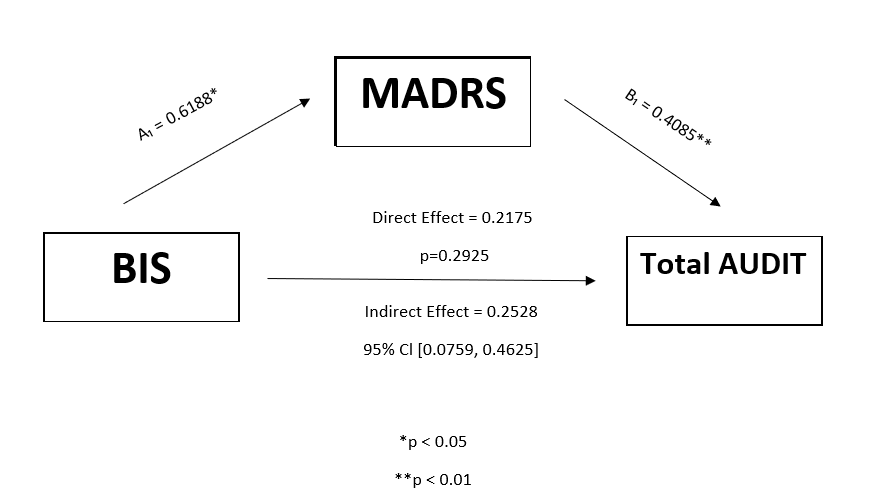


***Figure S4.*** *Single mediation with STAI as a mediator between BIS and Total OCDS (controlling for age)*


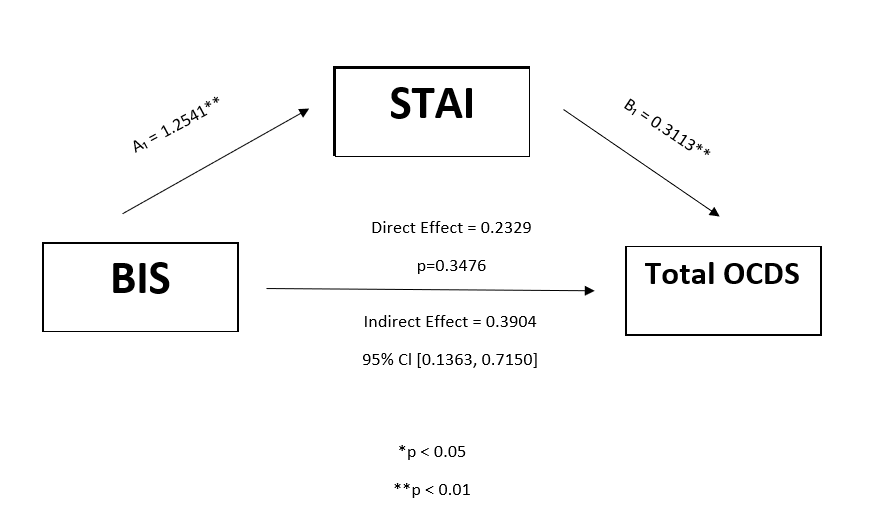


***Figure S5.*** *Single mediation with MADRS as a mediator between BIS and Total OCDS*


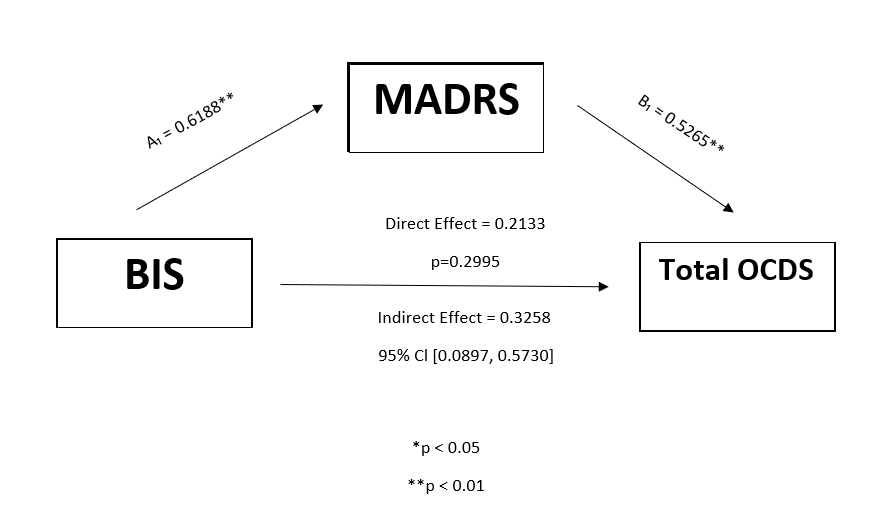

Supplement: Supplementary file 1 — Supporting Materials: brb370944‐sup‐0001‐SuppMatt.docx [file BRB3-15-e70944-s001.docx]
